# Fatal BK polyomavirus-associated pneumonia: report of two cases with literature review

**DOI:** 10.1186/s12879-023-08577-2

**Published:** 2023-09-11

**Authors:** Yuchen Wang, Yiling Fang, Ziyan Yan, Renfei Xia, Wenli Zeng, Wenfeng Deng, Jian Xu, Xiaoqin Feng, Jie Peng, Yun Miao

**Affiliations:** 1 Department of Transplantation, Nanfang Hospital, Southern Medical University, 1838 North Guangzhou Avenue, Guangzhou, 510515 China; 2 Department of Pediatrics, Nanfang Hospital, Southern Medical University, 1838 North Guangzhou Avenue, Guangzhou, 510515 China; 3 Department of Infectious Diseases, Nanfang Hospital, Southern Medical University, 1838 North Guangzhou Avenue, Guangzhou, 510515 China

**Keywords:** BK polyomavirus, Immunocompromised patients, Pneumonia, Hematopoietic stem cell transplantation

## Abstract

**Background:**

In immunocompromised populations, such as patients with AIDS and recipients of solid organ and hematopoietic stem cell transplants, BK polyomavirus (BKPyV) can reactivate and cause several diseases, which can lead to death in their severe forms. Unlike hemorrhagic cystitis and BKPyV-associated nephropathy, BKPyV-associated pneumonia is rare, with only seven known cases worldwide. However, the disease can rapidly progress with extremely high mortality.

**Case presentation:**

Herein, we report two cases of BKPyV-associated pneumonia following hematopoietic stem cell transplantation. Both patients had consistent infectious pneumonia and graft-versus-host disease after stem cell transplantation. The diagnosis of BKPyV-associated pneumonia was confirmed by metagenomic next-generation sequencing and polymerase chain reaction after the sudden worsening of the pulmonary infection signs and symptoms concomitant with renal dysfunction and systemic immune weakening. Both patients eventually died of systemic multi-organ failure caused by severe pneumonia.

**Conclusions:**

Currently, BKPyV reactivation cannot be effectively prevented. Immunocompromised patients must actively manage their primary lung infections, pay close attention to pulmonary signs and imaging changes. Especially during and after steroid pulse therapy or immunosuppressive therapy for graft versus host diseases, BKPyV load in blood/urine needs to be regularly measured, and the immunosuppressive intensity should be adjusted properly after the BKPyV reactivation diagnosis. Clinical trials of new antiviral drugs and therapies for BKPyV are urgently needed.

## Background

BK polyomavirus (BKPyV) is a member of the *Polyomaviridae* family, and it was first identified in 1971 by electron microscopy of the urine and uroepithelial cells of a renal transplant recipient [1]. BKPyV has been suggested to spread via various transmission routes, including respiratory, fecal–oral, and bloodborne [2], and it is ubiquitous in the general population as a lifelong latent or asymptomatic infectious agent in immunocompetent individuals. Nevertheless, in immunocompromised patients, BKPyV can be reactivated and cause several diseases, including hemorrhagic cystitis and BKPyV-associated nephropathy, while in its severe forms, it can lead to BKPyV-associated tumors, graft loss in renal transplantation recipients, and even death [3–5].

Compared to hemorrhagic cystitis and BKPyV-associated nephropathy, BKPyV-associated pneumonia is a rare condition with a high mortality rate, with only seven cases reported worldwide (Table 1) [6–12]. This paper described two cases of BKPyV-associated pneumonia following stem cell transplantation at a single center and conducted a literature review. We also formulated a possible theory about the BKPyV-associated pneumonia etiology and presented an overview of the disease course and management required to reduce morbidity and improve the BKPyV-associated pneumonia prognosis.

**Table 1:** Characteristics of cases with BKPyV-associated pneumonia [3–9]

Case	Report Year	Age (years)/Sex	Underlying condition	Antiviral drugs	Clinical outcome	Survival (days)
1	1993	27, M	Hemophilia type A, AIDS	N/A	Death	N/A
2	1997	0.7, F	Osteopetrosis, CBT (4/6)	Acyclovir	Death	23
3	2000	14, M	AIDS	N/A	Death	5
4	2002	49, M	CLL	Acyclovir	Death	18
5	2005	69, F	CLL	N/A	Death	6
6	2012	39, M	ALL, PBSCT (mismatched)	Vidarabine; Cidofovir	Death	4
7	2018	70, F	DLBCL, auto-PBSCT	Cidofovir	Recovery	N/A
8	Our Case, 2023	6, M	β-thalassemia, PBSCT(6/6)	Ganciclovir; Cidofovir	Death	10
9	Our Case, 2023	19, M	ALL, PBSCT (haplo)	Cidofovir	Death	12

The term of survival is calculated from the day of BK polyomavirus-associated pneumonia onset

*M* Male, *F* Female, *AIDS* Acquired Immune Deficiency Syndrome, *CBT* Cord blood transplantation, *CLL* Chronic lymphocytic leukemia, *ALL* Acute lymphocytic leukemia, *PBSCT* Peripheral blood stem cell transplantation, *DLBCL* Diffuse large B-cell lymphoma, *N/A* Not available

## Case presentation

### Case 1

**Fig. 1:**
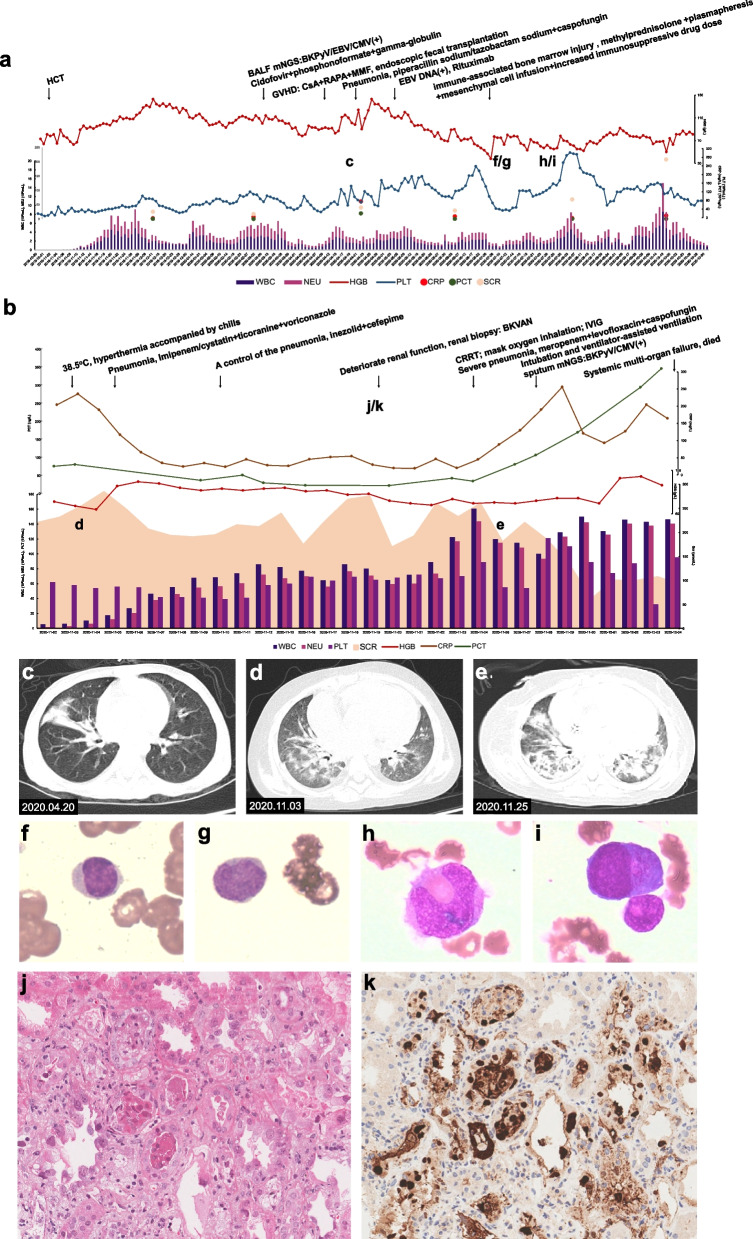
Clinical information of Case 1 from hematopoietic cell transplantation (HCT) to death. **a** The clinical course from HCT to admission. **b** The clinical course during the last hospitalization. **c**-**e** Images of the non-enhanced chest CT scans. **c** Inflammation of the posterior segment of the upper lobe and the lateral segment of the middle lobe, with patchy hyperintensity and vague margin. **d** Diffuse systemic inflammation in both lungs, with increased, thickened, and disorganized texture, multiple diffuse plaques, and patchy, nodular hyperintensities. **e** Diffuse systemic inflammation in both lungs that was significantly more progressive than in **d**. Lesions were located along the peribronchial sheath, showing fused patchy consolidation and ground-glass opacity, and high density in some nodules. **f**-**i** The bone marrow smear results. **f**, **g** Suppressed bone marrow proliferation, including for the granulocyte, erythroid, and macrophage lineages, with variable sizes of mature erythrocytes. Single platelets were rarely seen, indicating immune-mediated bone marrow failure. **h**, **i** Active bone marrow proliferation, with a granulocyte to erythrocyte ratio of 16.8:1. The monocyte proportion increased with no abnormalities in morphology, and platelets were relatively easily seen. **j**, **k** Pathology findings of BKPyVAN. **j** Hematoxylin and eosin (HE) staining. **k** Immunohistochemistry (IHC) against SV40-T. BALF, bronchoalveolar lavage fluid; BKPyV, BK Polyomavirus; CMV, cytomegalovirus; CRP, c reactive protein; CSA, cyclosporine A; EBV, Epstein–Barr virus; GVHD, graft versus host disease; HGB, hemoglobin; IVIG, intra-venous immunoglobulin; MMF, mycophenolate mofetil; mNGS, metagenomic next-generation sequencing; NEU, neutrophil count; PCT, procalcitonin; PLT, platelet count; RAPA, rapamycin; SCR, serum creatinine; WBC, white blood cell counts

**Fig. 2:**
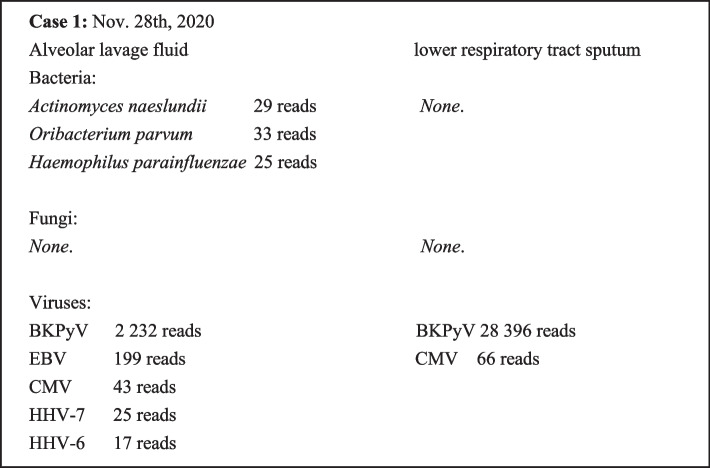
Detailed pathogens detected by mNGS in Case 1. BKPyV, BK Polyomavirus; CMV, cytomegalovirus; HHV, human herpes virus; EBV, Epstein–Barr virus

A male patient born in November 2014 was diagnosed with thalassemia major (βCD41-42 homozygote) on January 3, 2015. On October 29, 2019, the patient underwent allogeneic peripheral blood stem cell transplantation from a full match (6/6) unrelated donor (man, blood type O, aged 37) (Fig. 1a) at Nanfang Hospital, Southern Medical University. After transplantation, the patient tested positive for recurrent blood cytomegalovirus (CMV) and Epstein–Barr virus (EBV) infections, and on March 31, 2020, he underwent lung lavage because of bilateral pneumonia. The metagenomic next-generation sequencing (mNGS) analysis [13] of the lavage fluid DNA samples detected 2 232, 199, 43, and 25 specific sequences for BKPyV, EBV, CMV, and *Haemophilus parainfluenzae*, respectively. Meanwhile, his urinary BKPyV load was 3.25 × 10^9^ copies/mL, as shown by polymerase chain reaction (PCR). Therefore, he was placed on antiviral therapy with cidofovir, phosphonoformate, and intravenous gamma-globulin. On April 20, 2020, a chest CT scan showed poor pneumonia control that was resolved after the administration of piperacillin/tazobactam and caspofungin (Fig. 1c). Because the patient developed concomitant intestinal graft-versus-host disease (GVHD), cyclosporine, sirolimus, and mycophenolate mofetil were administered to maintain immunosuppression. To alleviate intestinal symptoms, recombinant anti-human tumor necrosis factor receptor 2 antibodies, budesonide, and thalidomide were administered, while vancomycin (orally) and endoscopic fecal transplantation were given to treat intestinal bacteria flora dysbiosis. At the May 3, 2020 follow-up, the patient’s EBV load was 5.17 × 10^3^ copies/mL, but it was cleared after six rounds of 0.1 g rituximab. Nevertheless, the bone marrow proliferation and differentiation potential were poor since the transplantation, with progressive hemoglobin (HGB) decline. On July 10, 2020, the bone marrow smear showed significant inhibition of cellular proliferation, including for the granulocytic, monocyte-macrophage, and erythroid lineages, while the mature erythrocytes had variable sizes (Fig. 1f and g), and single platelets were rare. Therefore, immune-mediated bone marrow failure was suspected, and the patient was treated with intravenous methylprednisolone infusion, increased immunosuppressive drug dose, plasmapheresis, and mesenchymal cell infusion. The treatment was temporarily effective (Fig. 1h and i), but the patient maintained high lactate dehydrogenase levels, and the glomerular filtration rate and platelet count steadily declined. On November 1, 2020, the patient presented with hyperthermia accompanied by chills and a peak temperature of 38.5 ℃; his post-admission blood test results (Fig. 1b) were as follows: routine blood test [white blood count (WBC) 0.74 × 10^9^/L, neutrophil count (NEU) 0.30 × 10^9^/L, HGB 81 g/L, platelet count (PLT) 78 × 10^9^/L], inflammatory markers [C-reactive protein (CRP) 37.94 mg/L, procalcitonin (PCT) 0.81 ng/L], renal function markers [serum creatinine (SCR) 182 μmol/ L], liver function markers [alanine aminotransferase (ALT) 28 U/L, aspartate aminotransferase (AST) 67 U/L, total bilirubin (TBIL) 3.9 μmol/L], and coagulation indices [activated partial thromboplastin time (APTT) 31.1 s, D-dimer 1.93 mg/L]. The urine test result showed 2 red blood cells/μL and it was positive for protein. Varicella-zoster virus (VZV), EBV, and CMV DNA were not detected in blood. Since a CT scan found diffuse multisystem inflammatory syndrome in both lungs, the patient received imipenem/cilastatin, ticoranine, and voriconazole as empirical therapy (Fig. 1d). A follow-up X-ray three days later showed control of pneumonia and inflammation absorption in both lungs, and the anti-infective regimen was changed to linezolid plus oral cefepime. Nevertheless, the patient’s renal function continued to deteriorate, accompanied by electrolyte imbalance; therefore, continuous renal replacement therapy (CRRT) was initiated. On November 19, 2020, a renal biopsy was performed, and the patient was diagnosed with BKPyV-associated nephropathy (Fig. 1j and k); the urinary and plasma BKPyV load levels were 3.05 × 10^11^ copies/mL and 2.56 × 10^8^ copies/mL, respectively. On November 25, 2020, the patient presented with shortness of breath and decreased oxygen saturation despite breathing oxygen through a face mask. A CT scan indicated severe pneumonia with a significantly worsened diffuse multisystem inflammatory syndrome in both lungs (Fig. 1e). Therefore, the anti-infective regimen was adjusted to meropenem plus levofloxacin and caspofungin with concomitant gamma-globulin infusion, and intubation and assisted ventilation were performed after 2 days. The mNGS analysis of the lower respiratory tract sputum samples revealed 28 396 (relative abundance 99.72%) and 66 (relative abundance 0.023%) specific sequences for BKPyV and CMV, respectively, while the PCR assay showed a BKPyV load of 4.02 × 10^9^ copies/mL. mNGS of alveolar lavage fluid also suggested dominant infection of BKPyV (detailed pathogens in mNGS are listed in Fig. 2). On December 3, 2020, a chest X-ray scan suggested worsening pneumonia with pneumomediastinum, ventricular flutter, and multiple organ system failure. The patient died on December 5, 2020, despite resuscitation.

### Case 2

**Fig. 3:**
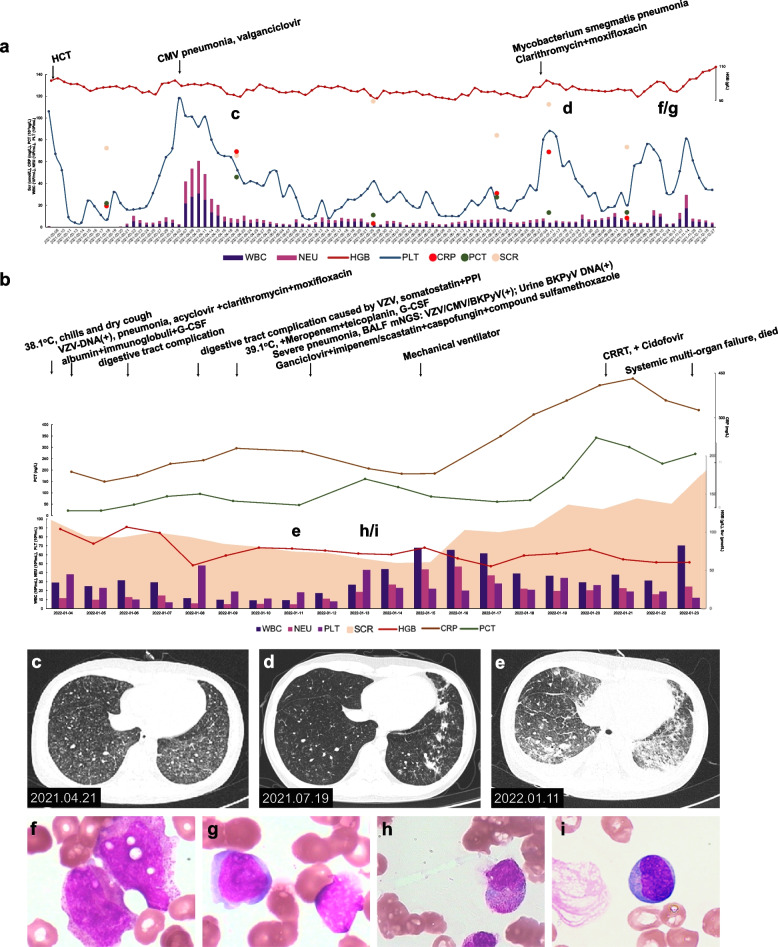
Clinical information of Case 2 from hematopoietic cell transplantation (HCT) to death. **a** The clinical course from HCT to admission. **b** The clinical course during the last hospitalization. **c**-**e** Images of the non-enhanced chest CT scans. **c** Mild chronic inflammation in the anterior basal segment of the lower lobe of both lungs and the external basal segment of the right inferior lobe. Multiple solid and ground-glass small nodules opacity were seen, with the lower lobe of both lungs as the most severe sites. **d** Systemic inflammation in both lungs, similar to **c**. **e** Systemic inflammation in both lungs characterized by multiple nodular, patchy, and solid plaque or ground-glass opacity, more advanced compared to **e**. **f**-**i** The bone marrow smear results before and after admission. **f**, **g** Active bone marrow proliferation, with a granulocyte to erythrocyte ratio of 0.73:1. Toxic granules were found in mature granulocytes, and platelets were rarely seen. **h**, **i** Suppressed bone marrow proliferation, with a granulocyte to erythrocyte ratio of 1.22:1. Megakaryocytes were not seen, and platelets were rarely seen. BALF, bronchoalveolar lavage fluid; BKPyV, BK Polyomavirus; CMV, cytomegalovirus; CRP, c reactive protein; CRRT, continuous renal replacement therapy; G-CSF, granulocyte colony-stimulating factor; HGB, hemoglobin; mNGS, metagenomic next-generation sequencing; NEU, neutrophil count; PCT, procalcitonin; PLT, platelet count; PPI, proton pump inhibitors; SCR, serum creatinine; VZV, varicella-zoster virus; WBC, white blood cell counts

**Fig. 4:**
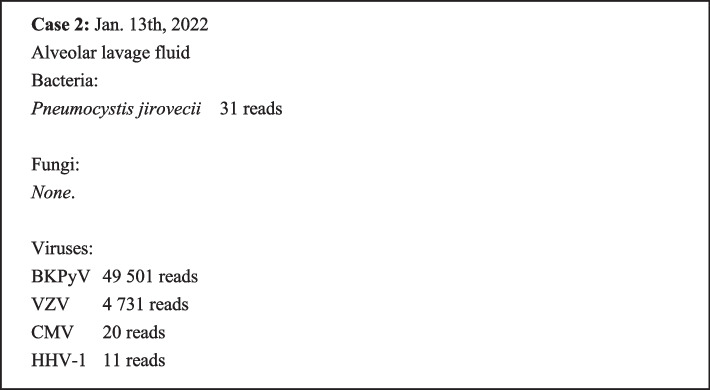
Detailed pathogens detected by mNGS in Case 2. BKPyV, BK Polyomavirus; CMV, cytomegalovirus; VZV, varicella-zoster virus; HHV, Human herpes virus

A male patient born in November 2002 was diagnosed with acute lymphoblastic leukemia in September 2020 and underwent paternal donor haploidentical allogeneic peripheral blood hematopoietic stem cell transplantation in March 2021 (Fig. 3), receiving regular postoperative anti-tumor therapy with sorafenib 0.8 g qd afterward. Nevertheless, the patient experienced recurrent postoperative pneumonia, and in April 2021, he developed CMV pneumonia (Fig. 3c) that improved after being treated with valganciclovir. Eventually, the patient tested negative for the virus. In July 2021, the patient had *Mycobacterium smegmatis* pneumonia (Fig. 3d), which improved after clarithromycin and moxifloxacin treatment, and the sputum culture was negative. The patient was placed on a long-term anti-infective maintenance therapy using the above regimen. The bone marrow examination on December 6, 2021 showed increased activity in the formation of red blood cells, poor platelet formation, and toxic granules in mature granulocytes (Fig. 3f and g). On January 2, 2022, the patient had a night fever of unknown origin with a temperature high of 38.1℃, accompanied by chills and a dry cough. The following day, a blister-like rash appeared all over the body, and some blisters burst, followed by pus and accompanied by pain. After admission (Fig. 3b), the blood test results were as follows: routine blood test (WBC 2.51 × 10^9^/L, NEU 0.98 × 10^9^/L, HGB 78 g/L, PLT 23 × 10^9^/L); inflammatory markers (CRP 86.11 mg/L and PCT 0.213 ng/L); renal function markers (SCR 94 μmol/L); liver function markers (ALT 167 U/L, AST 170 U/L, and TBIL 7.5 μmol/L); and coagulation indices (APTT 32.2 s and D-dimer levels 26.27 mg/L). The urine test result showed 118 red blood cells/mL and positive for protein. Blood VZV-DNA was positive while EBV, CMV, and herpes simplex virus were negative. A chest X-ray scan showed bilateral lower lobe pneumonia that was more severe in the left lobe. The bone marrow smear showed defects in the differentiation of megakaryocytes and platelets, with no abnormal residual leukocytes (Fig. 3h and i). After admission, the patient was administered acyclovir (a nucleoside analog) against VZV and clarithromycin (a macrolide antibiotic) combined with moxifloxacin (quinolone) against non-tuberculous mycobacteria. The patient also received the following medication: posaconazole, an inhibitor of the fungal lanosterol 14α-demethylase and a derivative of itraconazole, to prevent fungal infections; granulocyte colony-stimulating factor (G-CSF) to stimulate the maturation of various immune cells and accelerate leukopoiesis; eltrombopag, a thrombopoietin receptor agonist, to increase platelet count; magnesium isoglycyrrhizinate as an anti-inflammatory agent to protect the liver cell membrane and improve liver function; glutathione, an antioxidant and systemic detoxifier, for restoring the liver function; and albumin, immunoglobulin infusion, and nutritional support. On January 8, 2022, the patient’s HGB level decreased to 56 g/L, probably due to a digestive tract complication after varicella infection, and the symptoms were relieved after fasting and infusion of somatostatin and esomeprazole. On January 9, 2022, although his liver function improved, his body temperature increased to 39.5 °C, and the blood test suggested severe and progressing agranulocytosis, with low WBC and NEU levels of 0.98 and 0.48 × 10^9^/L, respectively. The inflammatory marker levels continued to rise, with CRP and PCT levels at 197.84 mg/L and 0.637 ng/L, respectively. Therefore, meropenem and teicoplanin were introduced, and the leukopoiesis treatment was intensified. On January 11, 2022, the blister-like rash gradually disappeared, but new red papules appeared on the limbs, and the new 24-h urine protein quantification level was 8.9 g. The patient gradually developed shortness of breath and a decreased oxygenation index despite breathing oxygen through a face mask. A chest CT scan showed an exacerbation of pneumonia, and combined viral, bacterial, and fungal infections were suspected (Fig. 3e). The results for the peripheral blood aerobic bacteria, anaerobic bacteria, and fungal cultures were negative. The mNGS analysis of alveolar lavage fluid suggested the presence of VZV, CMV, BKPyV, HHV-1 (human herpes virus-1), and *Pneumocystis jirovecii* (detailed pathogens in mNGS are listed in Fig. 4), and the BKPyV load in alveolar lavage fluid was 2.80 × 10^4^ copies/mL as shown by PCR. Meanwhile, the urinary BKPyV load was 9.50 × 10^6^ copies/mL, while plasma BKPyV was not detected. The anti-infective regimen was then adjusted to ganciclovir, imipenem/cilastatin, caspofungin, and compounded sulfamethoxazole. On January 15, 2022, the patient’s shortness of breath and dyspnea worsened, and he underwent tracheal intubation with assisted ventilation. Next, the patient experienced hyperpyrexia, progressively elevated infection indicators, hydrosarca, and acute renal failure. On January 20, 2022, CRRT was initiated with cidofovir antiviral therapy. Nevertheless, the patient’s condition deteriorated further with systemic multi-organ failure, and the patient died on January 23, 2022, despite resuscitation.

## Discussion and conclusions

A weakened immune system is the primary cause of BKPyV reactivation and various BKPyV-associated diseases [14]. BKPyV reactivation is commonly observed in immunosuppressed patients, such as stem cell- and solid organ transplant recipients, including renal transplant recipients. Due to the global increase in aging and advances in medical care, the immunocompromised patient fraction is increasing; therefore, BKPyV reactivation will occur more frequently as a complication of various underlying diseases [15, 16].

Although BKPyV has various transmission routes, current studies on its life cycle suggest that its main targets are renal tubular epithelial cells and urothelial cells [17]. The onset of BKPyV-associated diseases usually begins in the kidneys, ureters, and bladder, with viruria as the earliest BKPyV-associated manifestations and may gradually progress to BKPyV-associated nephropathy or hemorrhagic cystitis and possibly pneumonia [18]. The urothelium is also a suitable site for the long-term spread of BKPyV [19]. No primary onset BKPyV-associated pneumonia case has been reported so far and most cases were secondary to or combined with recent pulmonary infections of other bacteria, viruses (especially CMV and adenovirus), or fungi [17, 20–22]. BKPyV is sensitive to the immune status, and it may facilitate lytic and dominant BKPyV infections only in the context of local immune disorders and immune cell weakening caused by infections with other pathogens [23, 24].

The diagnosis of the previously reported cases of BKPyV-associated pneumonia relies mainly on a history of BKPyV viruria/DNAemia, hemorrhagic cystitis, or BKPyV-associated nephropathy [25], with lower sputum or alveolar lavage fluid pathogenic testing, and chest CT or radiographs. The radiological features of BK virus-associated pneumonia are similar to interstitial pneumonia and manifest as “ground-glass” changes. Histological features of lung tissue in autopsy include viral inclusion bodies within type II alveolar epithelial cells and positive immunohistochemistry staining against SV40-T in the cell nucleus. However, the limitation of the study lays in the lack of histological evidence and failure in the analysis of other respiratory RNA viruses by multiplex PCR, which can further enhance the clarity of diagnosis and improve the standardization of treatment.

Due to the insidious onset of the disease and clinicians’ lack of attention to BKPyV, the BKPyV blood/urine viral load is often already at a relatively high level when first detected. Furthermore, the occurrence of BKPyV-associated pneumonia indicates systemic immune weakening, comorbid systemic multi-organ dysfunctions especially with renal dysfunction, electrolyte disorders and acidosis, rapid progression, and poor prognosis. The mortality rate is as high as 88.9%, as the efficacy of antiviral drug administration is still uncertain so far and possibly imposes an additional burden on the patients’ liver and kidney functions, exacerbating liver and kidney failure.

In conclusion, BKPyV reactivation cannot be effectively prevented by now. Immunocompromised patients require active management of primary infections. Especially during and after steroid pulse therapy or immunosuppressive therapy for GVHD, regular tests of the BKPyV load in blood/urine should be performed. Primary lung infections must be managed in these patients, along with adjustment of immunosuppressive intensity, and targeted management of the BKPyV-associated urinary system diseases early on by local bladder administration may be considered. Additionally, close attention to pulmonary signs and imaging changes should be paid. In the long term, systematic clinical trials of antiviral drugs, such as immunoglobulins, cidofovir, and imipramine, and virus-specific T Cell therapy will help improve the prognosis of BKPyV-associated diseases.

## Data Availability

Not applicable.

## Abbreviations

ALT: Alanine aminotransferase
APTT: Activated partial thromboplastin time
AST: Aspartate aminotransferase
BKPyV: BK polyomavirus
CMV: Cytomegalovirus
CRP: C-reactive protein
CRRT: Continuous renal replacement therapy
EBV: Epstein–Barr virus
G-CSF: Granulocyte colony-stimulating factor
GVHD: Graft-versus-host disease
HGB: Hemoglobin
HHV-1: Human herpes virus-1
IVIG: Intra-venous immunoglobulin
mNGS: Metagenomic next-generation sequencing
NEU: Neutrophil count
PCR: Polymerase chain reaction
PCT: Procalcitonin
PLT: Platelet count
SCR: Serum blood creatinine
TBIL: Total bilirubin
VZV: Varicella-zoster virus
WBC: White blood count
